# Supplementary material for: Phytochemical Profile and In Vitro Bioactivities of Plant-Based By-Products in View of a Potential Reuse and Valorization
Source: Plants (Basel). 2023 Feb 10;12(4):795. doi: 10.3390/plants12040795 (PMC9961642; doi:10.3390/plants12040795)
Supplement: Supplementary file 1 [file plants-12-00795-s001.zip › plants-2150090-supplementary.pdf]

**Phytochemical profile and *in vitro* bioactivities of plant-based by-products in view of a potential reuse and valorization**

Ilaria Chiocchio,[a] Manuela Mandrone,[a*] Massimo Tacchini,[b] Alessandra Guerrini[b], Ferruccio Poli[a]

[a]Department of Pharmacy and Biotechnology, Alma Mater Studiorum – University of Bologna, Via Irnerio, 42 - 40126 Bologna, Italy

[b] Department of Life Sciences and Biotechnology, University of Ferrara, via Borsari 46, 44100 Ferrara, Italy.

***Correspondence**

Dr. Manuela Mandrone, University of Bologna, Department of Pharmacy and Biotechnology, Via Irnerio 42, 40126 Bologna, Italy

E-mail: manuela.mandrone2@unibo.it Phone: +390512091294; Fax +39051242576

## Supplementary material

**Table S1.** Total flavonoid content, total polyphenol content, *in vitro* antioxidant activity, anti-tyrosinase activity, and antibacterial activity (expressed as Minimum Inhibitory Concentration, MIC) of all samples.

| Scientific name | Sample tag | Total flavonoid content expressed in mg RE/g (DW) | Total polyphenol content expressed as mg GAE/g (DW) | Antioxidant activity expressed as mg Tr. Eq/mL of extract | Percentage of tyrosinase inhibition at 100 μg/mL | MIC *Clavibacter michiganensis* subsp. *nebraskense* (ATCC 27822) (mg/ml) | MIC *Pseudomonas syringae* pv *syringae* van Hall (ATCC 19310) (mg/ml) |
|---|---|---|---|---|---|---|---|
| ***Abutilon theophrasti* Medik.** | Ath | 13.73 ± 0.46 | 18.06 ± 0.57 | 0.95 ± 0.17 | 26 | >1.0 | >1.0 |
| ***Achillea millefolium* L.** | Acm | 14.54 ± 0.48 | 25.81 ± 0 | 0.85 ± 0.07 | 25 | >1.0 | >1.0 |
| ***Allium cepa* L.** | Ace | 1.36 ± 0.05 | 2.94 ± 0 | - | 0 | >1.0 | >1.0 |

| | | | | | | | |
|---|---|---|---|---|---|---|---|
| ***Artemisia absinthium* L.** | Ara | 16.18 ± 0.54 | 29.26 ± 3.16 | 0.89 ± 0.02 | 25 | >1.0 | >1.0 |
| ***Beta vulgaris* L.** | Bvu | 3.99 ± 0.13 | 6.32 ± 0.4 | - | 0 | >1.0 | >1.0 |
| ***Camelina sativa* (L.) Crantz** | Csa | 0.55 ± 0.02 | 2.48 ± 0.16 | - | 0 | >1.0 | >1.0 |
| ***Castanea sativa* Mill.** | Csp | 4.78 ± 0.16 | 43.97 ± 1.7 | 4.76 ± 0.16 | 84 | >1.0 | >1.0 |
| | Csr | 6.23 ± 0.21 | 30.73 ± 0.76 | 1.65 ± 0.11 | 20 | >1.0 | >1.0 |
| ***Cicer arietinum* L.** | Car | 7.92 ± 0.26 | 10.04 ± 0.56 | - | 20 | >1.0 | >1.0 |
| ***Cichorium intybus* L.** | Cia | 0.35 ± 0.01 | 4.67 ± 0.02 | - | 13 | >1.0 | >1.0 |
| | Cib | 18.86 ± 0.63 | 25.26 ± 1.39 | 1.22 ± 0.07 | 16 | >1.0 | >1.0 |
| ***Cucurbita pepo* L.** | Cpe | 13.21 ± 0.44 | 16.45 ± 0.23 | - | 0 | >1.0 | >1.0 |
| | Cpi | 19.88 ± 0.66 | 17.35 ± 1.1 | 0.71 ± 0.08 | 3 | >1.0 | >1.0 |
| ***Cupressus sempervirens* L.** | Css | 8.25 ± 0.27 | 36.39 ± 0.12 | 2.14 ± 0.11 | 41 | 1.0 | >1.0 |
| ***Echinochloa crus-galli* (L.) P.Beauv.** | Ecg | 16.4 ± 0.55 | 15.95 ± 1.2 | - | 2 | >1.0 | >1.0 |

| | | | | | | | |
|---|---|---|---|---|---|---|---|
| ***Erigeron canadensis* L.** | Eca | 36.12 ± 1.2 | 31.68 ± 0.16 | 1.75 ± 0.14 | 20 | >1.0 | >1.0 |
| ***Helianthus annuus* L.** | Han | 5.12 ± 0.17 | 13.66 ± 0.62 | - | 0 | >1.0 | >1.0 |
| ***Helichrysum italicum* (Roth) G. Don** | Hei | 41.19 ± 1.37 | 37.55 ± 0.55 | 2.58 ± 0.36 | 51 | 0.125 | >1.0 |
| ***Laurus nobilis* L.** | Lan | 12.79 ± 0.43 | 29.87 ± 1.01 | - | 10 | >1.0 | >1.0 |
| ***Lavandula angustifolia* Mill.** | Laa | 15.26 ± 0.51 | 37.52 ± 0.29 | 1.92 ± 0.07 | 22 | >1.0 | >1.0 |
| ***Melissa officinalis* L.** | Meo | 10.53 ± 0.35 | 36.49 ± 0.75 | 2.38 ± 0.46 | 25 | >1.0 | >1.0 |
| ***Origanum vulgare* L.** | Orv | 4.82 ± 0.16 | 18.01 ± 0.94 | 0.56 ± 0 | 20 | >1.0 | >1.0 |
| ***Phaseolus vulgaris* L.** | Pvb | 4.34 ± 0.14 | 6.11 ± 0.18 | - | 20 | >1.0 | >1.0 |
| | Pvu | 20.35 ± 0.68 | 19.04 ± 0.17 | 0.51 ± 0 | 13 | >1.0 | >1.0 |
| ***Prunus amygdalus* Batsch** | Pam | 3.66 ± 0.12 | 42.38 ± 0.31 | 5.1 ± 0.07 | 23 | >1.0 | >1.0 |
| ***Rosa damascena*** | Rod | 35.34 ± 1.18 | 43.96 ± 1.5 | 4.77 ± 0.12 | 22 | >1.0 | >1.0 |
| ***Salvia officinalis* L.** | Sco | 26.6 ± 0.89 | 38.53 ± 0.95 | 1.85 ± 0.11 | 46 | 0.5 | >1.0 |

| | | | | | | | |
|---|---|---|---|---|---|---|---|
| ***Salvia rosmarinus* Schleid.** | Sar | 22.9 ± 0.76 | 39.83 ± 0.43 | 2.03 ± 0.26 | 26 | 0.5 | >1.0 |
| ***Salvia sclarea* L.** | Sas | 17.03 ± 0.57 | 19.9 ± 0.37 | 0.48 ± 0.01 | 12 | 1.0 | >1.0 |
| ***Solanum lycopersicum* L.** | Sly | 19.35 ± 0.65 | 25.88 ± 0.4 | 1.06 ± 0.01 | 19 | >1.0 | >1.0 |
| ***Solanum tuberosum* L.** | Stu | 2.02 ± 0.07 | 5.98 ± 0.96 | - | 0 | >1.0 | >1.0 |
| ***Sorghum bicolor* (L.) Moench** | Sbl | 8.97 ± 0.3 | 16.12 ± 2.75 | 0.34 ± 0.01 | 0 | >1.0 | >1.0 |
| | Sbr | 2.78 ± 0.09 | 10.96 ± 0.01 | - | 10 | >1.0 | >1.0 |
| | Sbf | 1.41 ± 0.05 | 5.85 ± 0.08 | - | 3 | >1.0 | >1.0 |
| ***Thymus vulgaris* L.** | Tvu | 42.91 ± 1.43 | 42.05 ± 1.91 | 1.91 ± 0.03 | 20 | >1.0 | >1.0 |
| ***Triticum aestivum* L.** | Tae | 4.13 ± 0.14 | 5.95 ± 0.35 | - | 8 | >1.0 | >1.0 |
| ***Vitis vinifera* L.** | Vvi | 6.24 ± 0.21 | 29.36 ± 1.74 | 1.23 ± 0.02 | 10 | >1.0 | >1.0 |
| **Thymol** | | | | | | 0.0625 mg/ml | 0.0625 mg/ml |
| **Heliocuivre** | | | | | | 0.65625 μl/ml | 2.625 μl/ml |

**Table S2**. Semi-quantitative analysis of the identified metabolites by $^1$H NMR. Sar= *Salvia rosmarinus;* Sas= *Salvia sclarea*, Sco= *Salvia officinalis*, Css= *Cupressus sempervirens*

| Metabolite | Diagnostic $^1$H NMR Signal (δ) Used for the Quantification and Multiplicity | Number of Underlying Proton/s | Metabolite quantity in the dried plant material (μg/mg) | | | |
|---|---|---|---|---|---|---|
| | | | Sar | Sas | Sco | Css |
| **Rosmarinic acid** | 6.31, d | 1 | 19.72 | 2.96 | 23.35 | - |
| **Sclareol** | 0.86, s | 3 | - | 57.39 | - | - |
| **Shikimic acid** | 7.32, d | 1 | - | - | - | 2.67 |
